# Alzheimer’s and Hyperglycemia: Role of the Insulin Signaling Pathway and GSK-3 Inhibition in Paving a Path to Dementia

**DOI:** 10.7759/cureus.6885

**Published:** 2020-02-05

**Authors:** Nawar Muneer Aljanabi, Sahil Mamtani, Muthanna Mohammed Hasan Al-Ghuraibawi, Sunita Yadav, Lubna Nasr

**Affiliations:** 1 Internal Medicine, California Institute of Behavioral Neurosciences and Psychology, Fairfield, USA; 2 Infectious Diseases Research, Veterans Affairs Medical Center, Lebanon, USA; 3 Internal Medicine, Almustansiriyah University/College of Medicine, Baghdad, IRQ; 4 Research, Jacobi Medical Center, Bronx, USA; 5 Geriatrics, University of Miami Miller School of Medicine, Miami, USA

**Keywords:** alzheimer disease, insulin signaling pathway, type 3 diabetes, types 2 diabetes, insulin, insulin resistance

## Abstract

In this project, we are trying to review the articles that discuss the relationship between insulin signaling and Alzheimer's disease (AD). Another focus of this project is to find the best treatment regimen that can reduce the progression of AD in patients with impaired glucose metabolism. We used Pubmed database to collect our data and used the following keywords: Alzheimer’s disease, insulin signaling pathway, type 3 diabetes, type 2 diabetes, insulin, and insulin resistance in our revision; we included free articles that were published in the last 10 years and excluded articles that were written in any language other than English. We reviewed 68 articles. Forty-nine out of 68 articles were containing materials that are relevant for this project. We found that there is a relation between AD and the insulin signaling pathway. Insulin signaling pathway impairment leads to hyperphosphorylation of Tau protein, which plays a vital role in AD pathology. The effect of insulin on cognition is bidirectional; the intranasal route of insulin showed to have a promising effect on cognition improvement. Subcutaneous and intravenous insulin can increase the risk of dementia. Further studies are encouraged to use a specific anti-diabetic medication that can reduce the progression of AD.

## Introduction and background

Alzheimer's disease (AD) was first described in the second decade of the last century. However, during the last 30 years, clinicians have recognized the high mortality and morbidity rates associated with AD compared to other types of dementia [1]. Some 5.4 million Alzheimer's cases were reported in the United States (US) in 2016, of which, 96% of them are aged 65 and older. Three out of 10 people who are 85 years old or older have AD, while eight out of 10 AD patients are 75 years old or older. About 66% of AD patients in the US are females, and 33% of the patients are males [2]. As per the Alzheimer's association international conference, the number of AD patients in the US is 5.8 million, which roughly means that there are more than 400,000 new cases during the last three years [3]. This article aims to clarify the relation between insulin resistance and cognition impairment, focusing on AD, the most common form of cognitive impairment. Most of the clinicians are familiar with symptoms of AD and the functional impairment that corresponds to the limits imposed on the daily activities of these patients. Despite acknowledging the symptoms, the relation between AD and insulin receptor (IR) function and signaling pathway dysfunction is not entirely clear to most healthcare providers. In the following review article, we will simplify and present our understanding regarding this relationship and explain the pathophysiological association between AD and insulin signaling. In addition, we are trying to find if there is a preferred anti-diabetic regimen that can reduce the chance of getting AD or decrease the comorbidities of already diagnosed AD patients. By finding a management plan that can reduce the severity of AD symptoms, decrease the rate of functional decline or prevent the chance of developing AD altogether, we can effectively address the obstacles concerning morbidity, mortality, and increasing global financial costs of this debilitating condition.

## Review

We collected our data from the PubMed database. We have not followed the preferred reporting items for systematic reviews and meta-analysis guidelines in our study. We searched the databases using the following keywords: Alzheimer's disease, insulin signaling pathway, type 3 diabetes, type 2 diabetes, insulin, and insulin resistance. We reviewed free full-text studies in the English language only and excluded articles published in other languages. The vast majority of the studies that we searched for have been published in the last five years, and most of the articles were peer-reviewed articles. We followed the ethical and legal protocols while collecting our data.

Alzheimer’s disease

The first case of Alzheimer's disease (AD) was reported in 1907 by Alois Alzheimer [4]. It is one of the most common causes of dementia. Studies showed that five to seven out of 10 dementia cases are due to AD. This disease affects 44 million patients worldwide. Providing care for AD patients contributes to a significant portion of the healthcare budget for most of the countries. In the US, for example, AD care alone costs $600 million per year [5]. Dementia is one of the leading causes of death in England and Wales, as 11.6% of deaths were related to dementia [6]. AD can present in two forms: 1) sporadic form: this is the most common form and presents late in life. The sporadic form can happen due to environmental and genetic factors. The APO E gene is the most important genetic risk factor. Carriers for this gene have an odds ratio of 3 for AD and odds ratio of 12 if they are homozygous carriers. 2) On the other hand, the familial form of AD occurs due to a mutation in the following genes, amyloid precursor protein (APP), presenilin 1 (PSEN1), and presenilin 2 (PSEN2). This form of AD is not as common as sporadic, and its manifestations appear between the fourth and sixth decade of life [7-8]. AD is a complex and progressive neurodegenerative disorder [9]. One of the pathologic pathways that lead to AD starts with altered cleavage of APP, which produces insoluble amyloid-beta (Aβ) fibrils. Aβ fibrils diffuse into synaptic clefts and interfere with synaptic signaling. Later, oligo polymerizes and aggregates as plaques [10-11]. Two types of Aβ polymers are playing a vital role in the pathogenesis of AD: Aβ40 and Aβ42. Aβ42 is more neurotoxic than Aβ40 and Aβ40/Aβ42 accumulation results in ion channels blockage, calcium homeostasis disturbances, increased mitochondrial oxidative stress, reduced energy metabolism, and glucose regulation, contributing to the deterioration of neuronal health and neuronal cell death [12]. Another pathological mechanism includes the hyperphosphorylation of Tau proteins. Tau protein allows the organization and assembly of the microtubule, which facilitates stabilization. Hyperphosphorylated Tau proteins aggregate in the form of paired helical filaments, which is a significant component of the neurofibrillary tangles [13-15]. Memory impairment is the presenting symptom for most of the patients. Cognitive impairment may occur within or after the development of memory impairment. Late in the disease course, patients may develop executive dysfunction and visuospatial impairment. These deficits follow an insidiously progressive course [16]. Memory impairment in AD is unique. Episodic memory, especially memory for recent events that depend on the hippocampus and other medial temporal lobe structures is usually affected in AD patients. Procedural memory and motor learning rely on the subcortical system, which is not affected until late in the course of the disease [17-20]. As we mentioned above, AD follows a progressive disease course. This progression is measured with mental status scales. Studies showed that patients decline three points on average on the Mini-Mental State Examination each year. Additionally, less than 10% of AD patients have a rapidly progressive disease course, which includes five to six points decline on Mini-Mental State Examination each year [21-25]. Preclinical AD is a term used in the research world to define patients who have the structural and biochemical changes of AD, but they are not developing symptoms yet. Researches about interventions at this stage to reduce the progression to AD are encouraged [26]. Essential neuropathological changes in AD include amyloid beta-peptide deposition and neurofibrillary tangle formation due to neurofibrillary degeneration [27-28].

Insulin signaling

**Figure 1:**
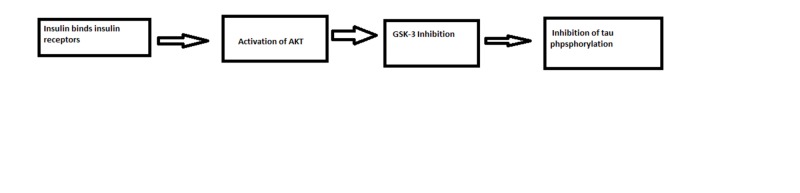
Physiology of insulin signaling that decrease tau protien toxicity. AKT: protein kinase B, GSK-3: glycogen synthase kinase 3

Insulin receptors are transmembrane receptors consisting of two extracellular alpha-subunits that are each attached to a beta-subunit. When insulin binds to the IR, it activates alpha-subunits and then activates beta-subunits of IR. Beta-subunits have tyrosine residues and also have a tyrosine kinase enzyme. Binding of insulin to the IR activates tyrosine kinase enzyme in the beta-subunits and leads to phosphorylation of the tyrosine residues. Following this, phosphorylation of the IR substrate (IRS) happens with the help of the tyrosine kinase enzyme of the beta subunits of the IR. Phosphorylation of the IRS will lead to conformational changes in its structure; these changes will attract another enzyme called phosphoinositide 3-kinases (PI3K), which is in the nonactive form to bind with the IRS. The binding between the IRS and the nonactive form of PI3K leads to the activation of the PI3K enzyme. Activation of PI3K enzyme will phosphorylate phosphatidylinositol (4,5)-bisphosphate (PIP2) in the cell membrane and leads to phosphatidylinositol (3,4,5)-trisphosphate (PIP3) formation. After this step, PIP3 formation facilitates protein kinase (AKT/PKB) activation. Active AKT has many activities which include the following: 1) Facilitate the binding of glucose transporter type 4 (GLUT4) receptors to the cell membrane, which leads to facilitate the entry of glucose and amino acids into the cell. 2) Increase glucokinase enzyme activity to start the glycolysis process. 3) Inhibition of glycogen synthase kinase 3 (GSK-3) enzyme, which leads to activation of glycogenesis through activation of glycogen synthase and inhibits GSK-3 induced tau hyperphosphorylation. 4) Lastly, phosphorylation of mTOR protein which increases lipids and protein synthesis in the cell. An intact insulin signaling pathway is essential to reduce the phosphorylation of tau protein and decrease the chance of getting AD [29]. Studies on rats showed that intracerebroventricular injection of streptozotocin, which is an IR inhibitor, would lead to long-term and progressive deficits in learning, memory, and cognitive behavior [30]. Rats injected with insulin had an enhancement in memory tasks in comparison with rats who were injected with normal saline [31]. In summary, insulin resistance leads to a decrease in the activity of AKT, which leads to inhibition of GSK-3, leading to hyperphosphorylation of tau protein, which is the main component of neurofibrillary tangles [32]. Figure 1 summarizes the role of insulin signaling in tau phosphorylation.

Diabetes mellitus and Alzheimer's disease

A study from Sackler school of medicine showed that there is a relation between hemoglobin A1c levels and performance on cognitive tests as the following, A 1% higher hemoglobin A1C level was associated with a 1.75 point lower on digit symbol substitution test score, a 0.20 point lower on Mini-Mental Status Examination score, a 0.11 point lower memory score, and a worse score on the Stroop Test [33]. Another study claimed that there is a decrease in cognition performance in patients who are having diabetic retinopathy [34]. While two other studies did not show a relation between diabetic retinopathy and cognitive decline, however in these studies, the retinal examination was minimal and did not include the entire retina, so the retinopathy was underestimated in these studies [35-36]. Maastricht Aging Study followed up the participants for 12 years and showed that participants with diabetes had a more significant decline in cognitive performance in comparison with participants without diabetes [37]. Also, acute hyperglycemia in type 2 diabetes patients has adverse effects on the speed of information processing and working memory [38]. Studies on an enzyme called insulin-degrading enzyme (IDE), which breaks down both insulin and Aβ peptide, showed that in hyperinsulinemia, IDE shifts towards degrading insulin more than Aβ peptide which leads to Aβ peptide accumulation [39-40]. Neurogenesis is the process where neuro-progenitor cells (NPCs) differentiate into different neuronal tissues. The neurogenesis process is affected negatively in the Goto-Kakizaki rat, a genetic model for type 2 diabetes [41]. Newly generated NPCs are usually labeled by bromodeoxyuridine. Nonobese diabetic mice model for type 1 diabetes showed a decrease in the number of labeled cells, which meant the decreased formation of new NPCs [42].

Anti-diabetic medication as a treatment option for Alzheimer's disease

Most of the available treatment options for AD are targeting the symptoms of the disease only, but they are not decreasing the progression of the disease. Glucagon-like peptide (GLP-1) receptors are present in the brain, where they have growth factor-like effects. These receptors also function to inhibit the programmed cell death of neuronal cells [43-44]. A study showed that mice with an abundance of GLP-1 receptors excess in the hippocampus were having increased neuronal growth rate and enhanced learning abilities [45]. The use of GLP1 agonists to reduce the chance of developing AD or decrease the severity of the symptoms of AD needs further investigations. The effect of insulin use on cognitive function is bidirectional. A study shows that there is a 50% increase in dementia risk in patients using peripheral insulin, which they attribute to the hypoglycemic effect of insulin [46]. Using intranasal insulin or using intravenous insulin under well-controlled conditions ensures a tight glycemic control which shows a promising effect in reducing the risk of developing dementia [47]. The effect of metformin on cognition is very controversial as one study showed that there is a protective effect of using metformin by reducing the deterioration rate of cognitive decline [48]. While other studies showed that there is no association between metformin intake and cognitive impairment [49]. Additional groups have shown that metformin intake harms cognition [46]. More studies are encouraged to determine the effect of sulfonylurea intake on cognition, as there are not enough studies available to determine the role of sulfonylurea [46].

## Conclusions

Alzheimer's dementia and diabetes mellitus are both conditions that are prominent in the developed world, adding to the healthcare costs and a rising trend of socioeconomic strife. Current research dictates novel strategies aimed to control or manage two birds with one stone. The use of GSK-3 inhibitors aimed at decreasing hyperphosphorylation and accumulation of tau proteins, a significant component of neurofibrillary tangles and memory impairment, is one such promising strategy, providing a new pathway to prevent or even regress the debilitating effects of Alzheimer's dementia. Other anti-diabetic medications also require further investigations in particular GLP-1 agonists, metformin, and insulin. Moreover, our future goals should be directed towards laying out specific pathways in GSK-3 inhibition in the brain, and an accurate model of Akt function, which will help better our understanding. Also, increasing the awareness of these intertwined molecular processes, including the relation between hyperglycemia, hyperinsulinemia, and the occurrence of AD, is essential to address as it will help patients and healthcare providers gain insight into this interesting association.
